# Myeloproliferative neoplasms with concomitant chronic myeloid leukemia are associated with TKI resistance and poor outcomes

**DOI:** 10.1038/s41375-026-02928-z

**Published:** 2026-03-27

**Authors:** Laura Li Gagnon, Andrea Duminuco, Fabio Stagno, Marta Sobas, Mikhail Fominykh, Andres Virchis, Franck-Emmanuel Nicolini, Julie Hildt, Helen Bentley, Anna L. Godfrey, Lee-Yung Shih, Ashlyn Chee, Adam Mead, Krzysztof Lewandowski, Patryk Sobieralski, Guy Hannah, Yasmin Reyal, Thomas Cummin, Jaroslaw Piszcz, Kawai Yip, Rebecca Frewin, Srinivasan Narayanan, Deborah Rahman, Alero Ajayi, Paul Cervi, Jennifer Byrne, Tom Taylor, Weronika Lebowa, Jiri Pavlu, Siamak Arami, Mansour Ceesay, Dominik Chraniuk, Robert Delage, Annalisa Condorelli, Clémence Santana, Deepti H. Radia, Hugues de Lavallade, Dragana Milojkovic, Claire Harrison, Patrick Harrington

**Affiliations:** 1 Department of Hematology, Guy’s and St Thomas’ NHS Foundation Trust, London, UK; 2 Department of Hematology, CHU de Québec – Université Laval, Québec, QC Canada; 3 Hematology Unit with BMT, A.O.U. Policlinico “G. Rodolico-San Marco”, Catania, Italy; 4 Division of Hematology, Department of Human Pathology in Adulthood and Childhood ‘Gaetano Barresi’, University of Messina, Messina, Italy; 5 Department and Clinic of Hematology, Cellular Therapies and Internal Medicine, Medical University of Wrocław, Wrocław, Poland; 6 Nicolaus Copernicus University Toruń, Ludwik Rydygier Collegium Medicum, Bydgoszcz, Poland; 7 Hematology Center LLP, Astana, Kazakhstan; 8 Semey Medical University, Semey, Kazakhstan; 9 Department of Hematology, The Royal Free London NHS Foundation Trust, London, UK; 10 Department of Hematology, University College London Hospitals NHS Foundation Trust, London, UK; 11 Department of Hematology, Leon Berard Cancer Center, Lyon, France; 12 Department of Hematology, Cambridge University Hospitals NHS Foundation Trust, Cambridge, UK; 13 School of Medicine, Chang Gung University, Taoyuan City, Taiwan; 14 Department of Internal Medicine, Division of Hematology-Oncology, Chang Gung Memorial Hospital at Linkou, Taoyuan City, Taiwan; 15 Department of Hematology, The Churchill Hospital, Oxford University Hospitals NHS Foundation Trust, Oxford, UK; 16 MRC Weatherall Institute of Molecular Medicine, NIHR, Biomedical Research Centre, University of Oxford, Oxford, UK; 17 Department of Hematology and Bone Marrow Transplantation, University of Medical Sciences, Poznań, Poland; 18 Department of Hematology and Transplantology, Medical University of Gdańsk, Gdańsk, Poland; 19 Department of Hematology, King’s College Hospital, London, UK; 20 Department of Hematology, St George’s University Hospitals NHS Foundation Trust, London, UK; 21 Department of Hematology, Portsmouth Hospitals University NHS Trust, Portsmouth, UK; 22 Department of Haematology, Internal Medicine and Angiology with Haematopoetic Cell Transplantation Unit, Medical University of Bialystok, Białystok, Poland; 23 Department of Hematology, Dartford and Gravesham NHS Trust, Dartford, UK; 24 Department of Hematology, Gloucestershire Hospitals NHS Foundation Trust, Gloucester, UK; 25 Department of Hematology, University Hospital Southampton NHS Foundation Trust, Southampton, UK; 26 Department of Hematology, Mid and South Essex NHS Foundation Trust, Essex, UK; 27 Department of Hematology, Southend University Hospital NHS Foundation Trust, Southend, UK; 28 Department of Hematology, Nottingham University Hospitals NHS Trust, Nottingham, UK; 29 Department of Hematology and Internal Medicine, Jagiellonian University Medical College, Kraków, Poland; 30 Department of Hematology, Hammersmith Hospital, Imperial College Healthcare NHS Trust, London, UK; 31 Department of Hematology, London Northwest Healthcare NHS Trust, London, UK; 32 Department of Hematology, Wojewódzki Szpital Zespolony w Toruniu, Toruń, Poland; 33 Divisione di Ematologia, ASST Papa Giovanni XXIII, Bergamo, Italy; 34 Hematology department, Centre Hospitalier de Valence, Valence, France; 35 School of Cancer and Pharmaceutical Science, King’s College London, London, UK

**Keywords:** Myeloproliferative disease, Epidemiology

## Abstract

Chronic myeloid leukemia (CML) and Philadelphia (Ph)-negative myeloproliferative neoplasms (MPN) are generally distinct clonal disorders, with the co-occurrence of *BCR::ABL1* rearrangement with concomitant Ph-negative MPN rarely reported. Here we describe the largest known international cohort of Ph-negative MPN and coexisting CML providing important insights into this rare clinical scenario. We performed an international, multicenter, retrospective analysis of patients with concomitant *BCR::ABL1* rearrangement and Ph-negative MPN, identifying 61 cases from 30 centers in 7 countries, over a 29-year period (1996–2025). Thirty-one patients (50.8%) had Ph-negative MPN preceding CML, 18 patients (29.5%) had CML preceding Ph-negative MPN, and 12 patients (19.7%) had Ph-negative MPN and CML diagnosed simultaneously. We observed increased TKI resistance and myelofibrotic transformation, especially in patients initially diagnosed with Ph-negative MPN. In this group, 35.4% (*n* = 11) progressed to MF, 2 patients to blast-phase MPN, and 69.2% (*n* = 18) failed to achieve a complete cytogenetic response. The rare e1a2 *BCR::ABL1* transcript was notably prevalent which is associated with TKI resistance and a more aggressive disease course in CML. We described superior survival in those with Ph-negative MPN preceding CML, with median OS not reached, compared with 277 months for CML preceding Ph-negative MPN and 100 months for those diagnosed simultaneously (*p* = 0.05).

## Introduction

Myeloproliferative neoplasms (MPN) are clonal disorders originating from hematopoietic stem cells with altered tyrosine kinase signaling, resulting in the proliferation of one or more myeloid lineages. They are classified into chronic myeloid leukemia (CML) and Ph-negative MPN. The translocation t(9;22) (q34.1;q11.2), which results in *BCR::ABL1* rearrangement, represents the pathognomonic molecular event of CML. Ph-negative MPN are sub-classified into polycythemia vera (PV), essential thrombocythemia (ET) and primary myelofibrosis (PMF) [1, 2]. Driver mutations in the Janus kinase 2 (*JAK2*) gene, most commonly at codon 617 (*JAK2 p.Val617Phe*), are found in nearly all patients with PV and in about 50-60% of ET and PMF cohorts. In a smaller proportion, mutations in the myeloproliferative leukemia (*MPL*) gene and calreticulin (*CALR*) gene are identified in ET and PMF.

*BCR::ABL1* rearrangement and MPN driver mutations, including *JAK2, CALR* and *MPL* mutations, were previously considered mutually exclusive. However, the rare occurrence of concomitant diagnosis of CML and Ph-negative MPN has been described in a limited number of case reports, single-center studies, or regrouped in systematic reviews [3–33]. The frequency of the combination of *BCR::ABL1* and *JAK2* mutation varies between 0.2% and 2.5% across different studies [7–10]. In the study by Martin-Cabrera et al., a total of 23 patients (0.2%) out of 10 875 MPN cases had evidence of both *JAK2* and *BCR::ABL1* [8]. Among more than 600 patients screened in a prospective single-center study, Hochman et al. identified 4 patients (0.67%) who were initially diagnosed with Ph-negative MPN and later developed CML [10]. The clonal evolution occurred between 10 and 36 years after the original diagnosis. The combination of *MPL* and *CALR* mutations with *BCR::ABL1* rearrangement is considerably rarer. Consequently, there is a lack of knowledge about this uncommon situation where *BCR::ABL1* rearrangement and MPN driver mutation coexist.

To address these uncertainties, we conducted an international, multicenter analysis of several cases of concomitant diagnosis of CML and Ph-negative MPN to improve our understanding of this rare scenario.

## Material and methods

### Study design and population

This was an international, multicenter, retrospective cohort study. Cases were identified following a survey of international experts. Patients were eligible if they had been previously diagnosed with an MPN and CML and had data on clinical presentation, molecular testing, therapeutic interventions, and outcomes.

### Data collection and methodology

Data were extracted from electronic medical records and institutional databases using a standardized template to ensure consistency across centers. Collected variables included demographics (age at diagnosis, sex, comorbidities), disease characteristics (MPN subtype, phase of CML at diagnosis, MPN driver mutation, additional mutations, cytogenetics, symptoms related to MPN, history of thrombosis, baseline blood counts, *BCR::ABL1* transcript variant, EUTOS long-term survival (ELTS)- score), time difference between diagnosis of CML and Ph-negative MPN, treatment details (drug regimens, modifications, lines of therapy), and outcomes (response to therapy, disease progression, survival). To minimize the variability, definitions of key variables were standardized.

The diagnosis of MPN and CML, the fibrotic transformation and progression to blast phase were defined according to the classification system in use at the time of diagnosis, ranging from the FAB classification to successive World Health Organization (WHO) classifications (2001, 2008, 2016, and 2022). This heterogeneity reflects the long study period (Supplementary Table 1). Response to treatment and disease phase definitions in CML were assessed based on *European LeukemiaNet* (ELN) 2020 recommendations for treating CML [34].

### Clonality analysis

Clonality analysis was performed in two cases from a single center as representative examples, based on local availability of material. A sequencing method was adopted to identify *JAK2* and *BCR::ABL1* clones in these two cases, following the technique of Khorashad JS et al. [35]. (Supplementary methods). We analyzed by sequential cloning two patients affected by CML who successively developed a Ph-negative MPN, and we were able to identify both *BCR::ABL1* and *JAK2* in distinct clones. Briefly, bone marrow samples were collected from patients following diagnosis after informed consent, and mononuclear cells were isolated by Ficoll-Paque Premium (GE Healthcare) density gradient centrifugation according to manufacturer’s protocol. cDNA was synthesized, subjected to real-time quantitative polymerase chain reaction and screened for *JAK2* and *BCR::ABL1* mutations by direct sequencing. In addition, amplified DNA fragments were cloned, plasmid DNA was extracted using SV Minipreps (Promega, Southampton, United Kingdom) and subsequently subjected to Sanger sequencing [35].

### Statistical analyses

Descriptive statistics were used to summarize baseline demographic and clinical characteristics: categorical variables were presented as frequencies and percentages, while continuous variables were reported as medians with interquartile ranges.

Exploratory survival analysis was performed with overall survival (OS) using the Kaplan-Meier method for the entire cohort, by group, and according to *BCR::*ABL1 transcript, ELTS score, driver mutation and MPN disease. OS was calculated as time from first diagnosis date to death or last follow-up. OS data were directly available for 44 of the 61 patients. For the remaining 17, follow-up information was extrapolated from available clinical data including laboratory and treatment follow-up data. The comparison of survival outcomes was performed using the log-rank Mantel Cox test. Progression-free survival (PFS) was analyzed for patients with ET, PV and MPN-U progressing to MF, using Kaplan-Meier estimates. Median PFS was calculated for the entire group, and separate survival curves were generated for each diagnostic subgroup. The time to onset of the second condition was compared using the Mann-Whitney U test. Mann-Whitney (for 2 variables) and Kruskal-Wallis (for 3 variables) analyses were also performed to compare whether different variables from each subgroup had an impact on prognosis, and p-values were calculated accordingly. A p-value less than 0.05 was considered significant. MedCalc Statistical Software version 19.2.6 (MedCalc Software bv, Ostend, Belgium; https://www.medcalc.org; 2020) and Prism 9 (version 9.5.1, 528, January 24, 2023) were used for statistical analysis.

## Results

### Study population and baseline characteristics

**Fig. 1:**
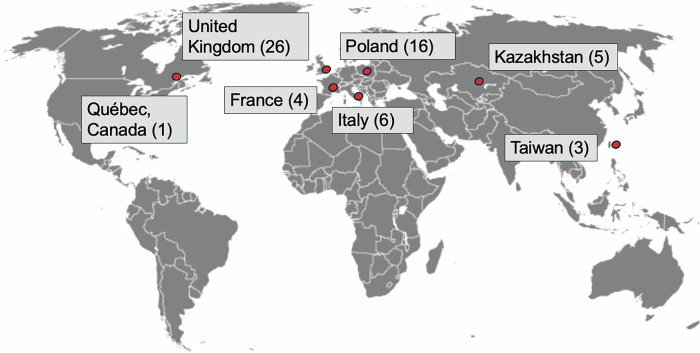
Geographical Distribution of Cases. Worl map showing the distribution of cases across the 7 participating countries.

Study and baseline characteristics are presented in Table 1. A total of 61 cases (50.8% female) across 30 centers in 7 countries, including United Kingdom, Poland, Italy, Kazakhstan, France, Taiwan and Canada (Fig. 1), were identified over a 29-year period (1996–2025). We divided the cohort into 3 groups: Group 1—Ph-negative MPN preceding CML (*n* = 31, 50.8%), Group 2—CML preceding Ph-negative MPN (*n* = 18, 29.5%), and Group 3—Ph-negative MPN and CML diagnosed simultaneously (*n* = 12, 19.7%).

**Table 1:** Study and Baseline Characteristics.

	Median Age at First Diagnosis (*y*) (range)	History of Thrombosis at any time (*n*, %)	Frequency of ET (*n*, %)	Frequency of PV (*n*, %)	Frequency of PMF (*n*, %)	Frequency of MPN-U (*n*, %)	Frequency of *JAK2* (*n*, %)	Frequency of *CALR* (*n*, %)	Frequency of MPL (*n*, %)
Group 1: Ph-negative MPN preceding CML 31/61 (50.8%)	59 (19–84)	6 (19.4%)	12 (38.7%)	9 (29.0%)	9 (29.0%)	1 (3.2%)	22 (75.9%)	5 (17.2%)	2 (6.9%)
Group 2: CML preceding Ph-negative MPN 18/61 (29.5%)	55 (34–81)	3 (16.7%)	8 (44.4%)	6 (33.3)	1 (5.6%)	3 (16.7%)	16 (88.9%)	2 (11.1%)	0
Group 3: Ph-negative MPN and CML diagnosed simultaneously 12/61 (19.7%)	57 (42–79)	3 (25.0%)	6 (50.0%)	1 (8.3%)	1 (8.3%)	4 (33.3%)	10 (83.3%)	1 (8.3%)	1 (8.3%)
Overall cohort (*n* = 61)	57 (19–84)	12 (19.7%)	26 (42.6%)	16 (26.2%)	11 (18.0%)	8 (13.1%)	48 (81.4%)	8 (13.6%)	3 (5.0%)

*MPN* myeloproliferative neoplasm, *CML* chronic myeloid leukemia, *Ph* Philadelphia, *ET* essential thrombocythemia, *PV* polycythemia vera, *PMF* primary myelofibrosis, *MPN-U* MPN-unclassifiable

### Group 1: Ph-negative MPN preceding CML

Most cases (*n* = 31, 50.8%) presented initially with Ph-negative MPN, including ET (*n* = 12, 38.7%), PMF (*n* = 9, 29.0%), PV (*n* = 9, 29.0%), and MPN-U (*n* = 1, 3.2%). There were no cases of prefibrotic MF. Median age at Ph-negative MPN diagnosis was 59 years (19–84). *JAK2* was detected in 70.9% (*n* = 22) of patients, whereas mutations in *CALR* and *MPL* gene in 16.1% (*n* = 5) and 6.5% (*n* = 2), respectively. Two patients (6.5%) were negative for recognized driver mutations but otherwise met MPN diagnostic criteria (Table 1). 19.4% (*n* = 6) had a history of thrombosis and 38.7% (*n* = 12) presented constitutional symptoms. Progression of ET/PV to myelofibrosis was noted in 35.4% (*n* = 11) of patients after a median of 10.5 years (2.7–20.0), and progression to blast-phase MPN in two patients (6.4%) after 8 months in one patient and 13 years in the other.

First-line treatment for Ph-negative MPN (Supplementary Table 2) included hydroxycarbamide (*n* = 21, 67.7%), ruxolitinib (*n* = 5, 16.1%), and anagrelide (*n* = 1, 3.2%). Four (12.9%) patients were managed with active surveillance, including venesections for PV individuals. Seventeen patients (54.8%) received second-line therapy, such as ruxolitinib (*n* = 9, 52.9%), hydroxycarbamide (*n* = 3, 17.6%), pegylated interferon (*n* = 2, 11.8%), anagrelide (*n* = 2, 11.8%), and momelotinib (*n* = 1, 5.9%).

Detection of *BCR::ABL1* was observed after a median of 121.5 months (5.0–318.2). In most cases, additional molecular testing was prompted by lack of treatment response and persistent or unexplained leukocytosis. At presentation, 28 patients were in chronic phase (CP-CML) and 3 in accelerated phase (AP-CML. The ELTS score was high for 46.4% (*n* = 13), intermediate for 32.1% (*n* = 9), and low for 21.4% (*n* = 6) of the 28 CP-CML patients. Common and rare *BCR::ABL1* transcript subtypes (Table 2) were detected in the Group 1: e13a2 (*n* = 12, 44.4%), e14a2 (*n* = 10, 37.0%), e1a2 (*n* = 4, 14.8%), and e19a2 (*n* = 1, 3.7%). Of 11 patients with myelofibrotic transformation, 2 patients had e1a2 subtype. Mutations in additional genes/non-driver mutations, including mutation in *ASXL1* (*n* = 5), *PHF6* (*n* = 2), *SF3B1* (*n* = 2), *TET2* (*n* = 4), *DNMT3A* (*n* = 2) and *IDH2* (*n* = 1), were detected in 9 different patients over the 21 tested. Among the 9 patients with additional mutations, 6 were identified at the time of initial diagnosis of CML, MPN, or concomitant diagnosis. In the remaining 3 patients, additional mutations were identified at the time of disease progression.

**Table 2:** Distribution of *BCR::ABL1* Fusion Transcripts in the 3 Groups.

	Common *BCR::ABL1* transcripts		Rare *BCR::ABL1* transcripts		
	e13a2 (*n*, %)	e14a2 (*n*, %)	e1a2 (*n*, %)	e19a2 (*n*, %)	e6a2 (*n*, %)
Group 1: Ph-negative MPN preceding CML 31/61 (50.8%) Available data 27/31 (87.1%)	12 (44.4%)	10 (37.0%)	4 (14.8%)	1 (3.7%)	0
Group 2: CML preceding Ph-negative MPN 18/61 (29.5%) Available data 12/18 (66.7%)	5 (41.7%)	5 (41.7%)	2 (16.6%)	0	0
Group 3: Ph-negative MPN and CML diagnosed simultaneously 12/61 (19.7%) Available data 12/12 (100%)	7 (58.3%)	3 (25.0%)	1 (8.3%)	0	1 (8.3%)
Overall cohort (*n* = 61) Available data 51/61 (83.6%)	24 (47.1%)	18 (35.3%)	7 (13.7%)	1 (1.9%)	1 (1.9%)

*MPN* myeloproliferative neoplasm, *CML* chronic myeloid leukemia, *Ph* Philadelphia.

First-line treatment for CML included imatinib (*n* = 25, 80.6%), dasatinib (*n* = 2, 6.5%), and nilotinib (*n* = 4, 12.9%). Among patients with follow-up data, median duration of first-line TKI was only 9 months (0.7–123). Response data were available for 26/31 patients (83.9%) (Table 3): 26.9% (*n* = 7) did not achieve complete hematological response (CHR), and 42.3% (*n* = 11) did not achieve complete cytogenetic response (CCyR). However, 15.4% (*n* = 4) achieved major molecular response (MMR), and 15.4% (*n* = 4) achieved deep molecular response (DMR). Of the 11 patients with ≥12 months on their first TKI, 4 obtained DMR, 3 MMR, 2 less than CCyR, 1 less than CHR, and 1 had no *BCR::ABL1* monitoring. Twelve patients (38.7%) received second-line treatment for CML, including dasatinib (*n* = 5, 41.7%), bosutinib (*n* = 3, 25.0%), nilotinib (*n* = 2, 16.7%), imatinib (*n* = 1, 8.3%), and ponatinib (*n* = 1, 8.3%). Six of these 12 patients were prescribed a third-line therapy for CML, including asciminib (*n* = 3), bosutinib (*n* = 1), dasatinib (*n* = 1), and imatinib (*n* = 1).

**Table 3:** First-Line TKI Best Response among Patients with Follow-up Data in the 3 Groups.

	No CHR (*n*, %)	No CCyR (*n*, %)	CCyR / *BCR::ABL1* < 1% IS (*n*, %)	MMR (*BCR::ABL1* < 0.1% IS) (*n*, %)	DMR (*BCR::ABL1* <0.01% IS) (n, %)
Group 1: Ph-negative MPN preceding CML 31/61 (50.8%) Available data 26/31 (83.9%)	7 (26.9%)	11 (42.3%)	0	4 (15.4%)	4 (15.4%)
Group 2: CML preceding Ph-negative MPN 18/61 (29.5%) Available data 17/18 (94.4%)	3 (17.6%)	1 (5.9%)	4 (23.5%)	3 (17.6%)	6 (35.3%)
Group 3: Ph-negative MPN and CML diagnosed simultaneously 12/61 (19.7%) Available data 8/8 (100%)	2 (25.0%)	2 (25.0%)	0	2 (25.0%)	2 (25.0%)
Overall cohort (*n* = 61) Available data 51/57 (89.5%)	12 (23.5%)	14 (27.5%)	4 (7.8%)	9 (17.6%)	12 (23.5%)

*MPN* myeloproliferative neoplasm, *CML* chronic myeloid leukemia, *Ph* Philadelphia, *TKI* Tyrosine Kinase Inhibitors, *CHR* complete hematological response, *CCyR* complete cytogenetic, *MMR* major molecular response, *DMR* deep molecular response.

In Group 1, MPN-directed therapy was continued at the time of CML diagnosis in 16 patients and combined with TKI therapy. Specifically, 5 patients received a TKI combined with hydroxycarbamide, 2 received a TKI combined with pegylated interferon, 8 received a TKI combined with ruxolitinib, and 1 patient received a TKI combined with momelotinib.

Two of the 6 patients who were tested for kinase domain (KD) mutations had KD mutations detected. One patient with *A433T, L387F, M244V*, and *A337T* mutations had received prior treatment with imatinib, bosutinib and then asciminib. The second patient with a *T315I* mutation was treated initially with imatinib for 4 months, prior to switching to ponatinib for 35 months, and is now on asciminib, achieving MMR. Three patients progressed to advanced phase CML (2 AP, 1 blast phase) on TKI after a median of 72 months (15–128).

### Group 2: CML preceding Ph-negative MPN

A smaller subgroup of 18 patients (29.5%) developed Ph-negative MPN after an initial CML diagnosis of which 17 were in CP-CML and 1 AP-CML. Median age at CML diagnosis was 54 years (34–81). The *BCR::ABL1* transcript subtypes identified in this group included e13a2 (*n* = 5, 41.7%), e14a2 (*n* = 5, 41.7%), and e1a2 (*n* = 2, 16.6%) (Table 2).

Median time from CML diagnosis to diagnosis of ET (*n* = 8, 44.4%), PV (*n* = 6, 33.3%), MPN-U (*n* = 3, 16.7%), and PMF (*n* = 1, 5.6%) was 55.6 months (3.6-168.1) (Table 1). There was significantly longer time to onset of the second condition in patients with Ph-negative MPN preceding CML (Group 1, median 121.5 months) compared with CML preceding Ph-negative MPN (Group 2, median 55.6 months, *p* = 0.049). Additional molecular testing was requested due to an inadequate response to treatment or unexpected disease progression with atypical features, such as persistent thrombocytosis or splenomegaly in CML. *JAK2* (*n* = 16, 88.9%) and *CALR* (*n* = 2, 11.1%) gene mutations were detected. Three cases (16.7%) transformed to myelofibrosis, after a median of 10.7 years (6.0–12.9) from the initial Ph-negative MPN diagnosis.

Regarding CML treatment, all patients (*n* = 18) received imatinib as first-line TKI for a median of 31 months (5-259). Response data were available for 17/18 patients (94.4%) (Table 3): 3 patients (17.6%) did not achieve CHR, and 1 (5.9%) did not achieve CCyR. In contrast, 4 (23.5%) achieved CCyR, 3 (17.6%) achieved MMR, and 6 (35.3%) achieved DMR. Most of the group (*n* = 15, 83.3%) completed ≥12 months treatment with imatinib. Nine patients (50.0%) switched to a second-line TKI (5 dasatinib and 4 nilotinib) for different reasons including resistance, loss of response, and significant side effects. Five of these 9 patients on a second-line TKI received a third-line TKI (2 ponatinib, 1 nilotinib, 1 bosutinib, and 1 dasatinib) and achieved DMR (*n* = 4) and CCyR (*n* = 1). All 5 patients in this group with *BCR::ABL1* KD mutation data had a negative analysis result, but *DNMT3A* was detected in two patients and *ASXL1* in a single patient.

First-line treatment for Ph-negative MPN (Supplementary Table 2) included hydroxycarbamide (*n* = 10, 55.6%), ruxolitinib (*n* = 2, 11.1%), and pegylated interferon (*n* = 1, 5.5%). 5 (27.8%) patients were managed with active surveillance, including venesections for PV individuals. Five patients (27.8%) received second-line therapy, such as hydroxycarbamide (*n* = 1), ruxolitinib (*n* = 2), and anagrelide (*n* = 2).

In Group 2, 12 of 18 patients continued TKI therapy at the time of MPN diagnosis. Combination therapy consisted of hydroxycarbamide in 10 patients, pegylated interferon in 1 patient, and ruxolitinib in 1 patient.

### Group 3: Ph-negative MPN and CML diagnosed simultaneously

Twelve patients (19.7%) were simultaneously diagnosed with CP-CML and Ph-negative MPN, with a median age of 57 years (42–79). MPN subtypes were ET (*n* = 6, 50.0%), MPN-U (*n* = 4, 33.3%), PMF (*n* = 1, 8.3%), and PV (*n* = 1, 8.3%), with *JAK2* (*n* = 10), *CALR* (*n* = 1), and *MPL* (*n* = 1) as driver mutations (Table 1). *BCR::ABL1* transcript subtypes were e13a2 (*n* = 7, 58.3%), e14a2 (*n* = 3, 25.0%), e1a2 (*n* = 1, 8.3%), and e6a2 (*n* = 1, 8.3%) (Table 2). One patient with e1a2 *BCR::ABL1* transcript, progressed to CML-AP after 39 months.

Regarding CML treatment, 8 out of 12 patients received a first-line TKI (7 imatinib and 1 dasatinib) and were treated for a median of 44 months (1–149). Three patients were not offered TKI but instead started Ph-negative MPN treatment. Another patient opted for palliative care. First-line treatment for Ph-negative MPN included hydroxycarbamide (*n* = 5, 41.7%), active surveillance, including venesections for PV patients (*n* = 5, 41.7%), and ruxolitinib (*n* = 1, 8.3), while 1 patient (8.3%) received palliative care (Supplementary Table 2). Three patients received combination therapy with a TKI and hydroxycarbamide, and 1 patient received a TKI combined with ruxolitinib. The remaining patients were treated with either tyrosine kinase inhibitor therapy alone or MPN-directed therapy alone.

Response data were available for the 8 patients who received a first-line TKI: 2 patients did not achieve CHR, and 2 did not achieve CCyR. However, 2 achieved MMR, and 2 achieved DMR (Table 3). Six out of 8 patients completed ≥12 months on their first-line TKI. Only 1 patient switched to a second-line TKI, dasatinib. Only one patient in this group had KD mutation data, and the result was negative. *DNMT3A* and *TET2* were detected once each in 2 different patients.

### Prognostic outcomes

**Fig. 2:**
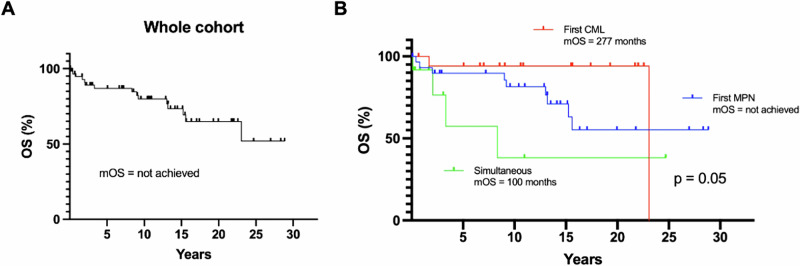
Overall survival (OS) for the whole cohort and Groups 1–3. OS for the entire cohort (**A**) and by group (**B**). MPN: myeloproliferative neoplasm, CML: chronic myeloid leukemia.

**Fig. 3:**
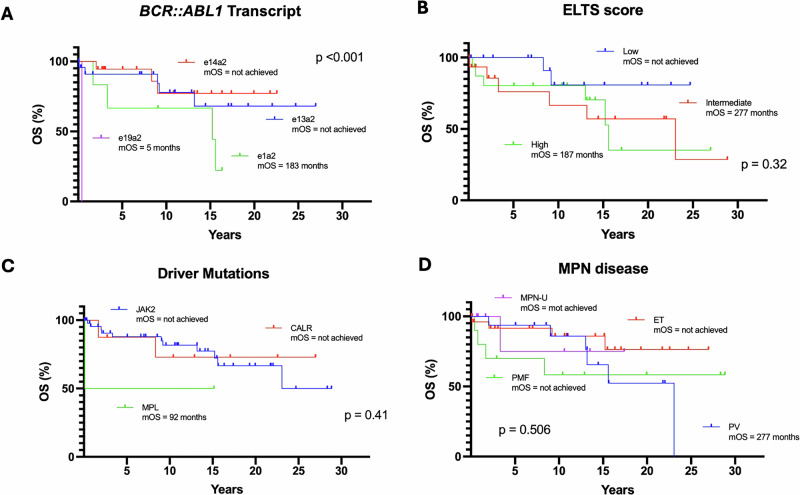
Overall survival (OS) according to *BCR::ABL1* transcript, ELTS score, driver mutations and MPN disease. OS according to BCR::ABL1 transcript (**A**), ELTS score (**B**), driver mutations (**C**), and MPN disease (**D**). MPN: myeloproliferative neoplasm, CML: chronic myeloid leukemia. ET: Essential thrombocythemia, PV: Polycythemia vera, PMF: Primary Myelofibrosis, MPN-U: MPN-unclassifiable.

The median OS for the entire cohort was not reached at the time of analysis (Fig. 2A). When stratified by diagnostic sequence groups (Fig. 2B), patients with Ph-negative MPN preceding CML (Group 1) demonstrated the most favorable survival, with a median OS not reached after a median follow-up of 156 months (1–346) (31 patients, 9 deaths). The median follow-up from the diagnosis of CML was 33.4 months (range 1–157.2 months). In contrast, those with CML preceding Ph-negative MPN (Group 2) had a median OS of 277 months (18 patients, 2 deaths), and those with Ph-negative MPN and CML diagnosed simultaneously (Group 3) had the shortest median OS of 100 months (12 patients, 4 deaths) (*p* = 0.05), after a median follow-up of 129 (8–277) and 22 months (1–296), respectively. Additionally, OS analysis stratified by *BCR::*ABL1 transcript type demonstrated a statistically significant difference (*p* < 0.001) (Fig. 3A), with inferior outcome with e1a2 transcript. However, it should be noted that only one patient carried the e19a2 transcript and one the e6a2 transcript, with the latter censored due to a very short follow-up of 2 months. OS analyses according to ELTS score, driver mutation, and MPN disease, did not reveal statistically significant differences (Fig. 3).

**Fig. 4:**
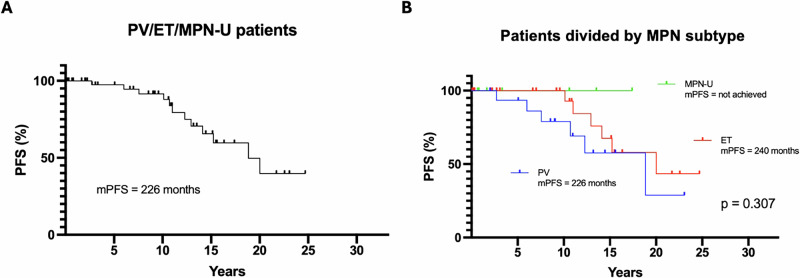
Progression-free survival (PFS) for patients with ET, PV and MPN-U progressing to myelofibrosis. PFS for patients with ET, PV and MPN-U combined (**A**) and divided between MPN subtype (**B**). ET: Essential thrombocythemia, PV: Polycythemia vera, MPN-U: MPN-unclassifiable.

PFS, defined as time to transformation to MF, was analyzed for patients with PV, ET, or MPN-U (*n* = 50). The combined median PFS for these patients was 226 months, with 12 transformations observed (Fig. 4A). When evaluated separately (Fig. 4B), median PFS differed across subtypes: patients with PV (*n* = 16) experienced 6 transformations, those with ET (*n* = 26) had also 6 transformations, and none of the patients with MPN-U (*n* = 8) progressed. However, these differences were not statistically significant (*p* = 0.307). Three patients proceeded to allogeneic stem cell transplantation, with all alive at last follow-up, with no evidence of relapse.

In exploratory comparisons of prognostic factors, both median age at first diagnosis (*p* = 0.001) and *BCR::ABL1* transcript p190 (*p* = 0.05) showed a significant association with shorter overall survival (Table 4).

**Table 4:** Analysis for potential prognostic factors.

	Status after a median follow-up of 127 months N (%)		
	Censor	Uncensor	*p*
Median age at first diagnosis:			
− <58 years	29 (48)	2 (3)	0.001
− ≥58 years	13 (21)	17 (28)	
Sex:			
− Male	23 (38)	7 (11)	>0.999
− Female	23 (38)	8 (13)	
Cytogenetics (available for 27 patients):			
− Normal	15 (56)	2 (7)	0.326
− Abnormal	7 (20)	3 (11)	
MPN diagnosis:			
− PV, ET, MPN-U	39 (64)	11 (18)	0.439
− MF	4 (7)	7 (11)	
Driver MPN mutations (available for 59 patients):			
− *JAK2*	37 (63)	11 (19)	>0.999
− *CALR, MPL*	8 (13)	3 (5)	
Additional mutations (available for 43 patients):			
− No	20 (46)	12 (28)	0.129
− Yes	10 (23)	1 (3)	
*BCR::ABL1* transcript (available for 49 patients):			
− p190	4 (8)	3 (6)	0.05
− p210	34 (69)	8 (17)	
ELTS score at diagnosis (available for 49 patients):			
− Low	14 (29)	2 (5)	0.227
− Intermediate	10 (20)	6 (12)	
− High	11 (22)	6 (12)	
MPN treatment:			
− W&W/venesection	10 (16)	1 (2)	0.264
− At least 1 therapy	36 (59)	14 (23)	
CML treatment (TKI):			
− 1 line	28 (46)	12 (20)	0.344
− 2 lines	8 (13)	2 (3)	
− 3 lines	10 (16)	1 (2)	

*MPN* myeloproliferative neoplasm, *CML* chronic myeloid leukemia, *ET* essential thrombocythemia, *PV* polycythemia vera, *PMF* primary myelofibrosis, *MPN-U* MPN-unclassifiable, *TKI* tyrosine kinase inhibitors, *ELTS* EUTOS long-term survival.

### Clinical results from clonality analysis

Clonality analysis allowed us to detect distinct clonal populations in two patients with CML preceding MPN (one clone *BCR::ABL1* and a second clone *JAK2*). The first case was a 70-year-old male with a diagnosis of CML in chronic phase, (Ph-positive, *BCR::ABL1* positive: e14a2), and was treated with imatinib frontline, soon achieving an MMR. After 3 years of treatment, he exhibited clinical signs of accelerated phase, including splenomegaly, although he remained in MMR. Molecular re-evaluation showed the emergence of a *JAK2*-positive clone, and a bone marrow biopsy revealed the presence of an MPN-U.

The second case was a 55-year-old female patient with a diagnosis of CML in chronic phase, ELTS not determined at diagnosis (Ph-positive, *BCR::ABL1* positive: e14a2). She received first-line therapy with imatinib, obtaining an MMR. Five years later, while in DMR, she presented with thrombocytosis, and CML disease restaging showed the emergence of a molecular *JAK2*-positive clone. Bone marrow biopsy revealed the presence of an MPN.

In both patients, we analyzed by sequential cloning as described in Methodology and were able to identify *BCR::ABL1* and *JAK2* in separate clones.

## Discussion

This multicenter, retrospective study describes, to our knowledge, the largest international cohort of Ph-negative MPN and coexisting CML. It provides valuable insights into disease presentation, treatment response and clinical outcomes in patients with this rare clinical scenario. The inclusion of data from 7 countries provides a comprehensive overview of this uncommon genetic combination across varied clinical practices and healthcare systems. We reveal an increased incidence of TKI resistance and myelofibrotic transformation, especially in patients who were diagnosed initially with Ph-negative MPN followed by CML. In this group, 35.4% (*n* = 11) progressed to MF after a median of 10.5 years (2.7-20.0), two patients (6.4%) progressed to blast-phase MPN within 8 months in one patient and 13 years in the other, and 69.2% (n = 18) failed to achieve a CCyR, including 26.9% (*n* = 7) who did not reach CHR. The e1a2 *BCR::ABL1* transcript was notably prevalent and is known to be associated with TKI resistance and a more aggressive disease course in CML.

Our findings are consistent with previous studies demonstrating that most cases with a concomitant diagnosis of CML and Ph-negative MPN, involve Ph-negative MPN preceding CML. Zanelli et al. performed a systematic review on the co-occurrence of *JAK2* mutation and *BCR::ABL1* translocation and identified Ph-negative MPN preceding CML in 49.4% (43/87), CML and Ph-negative MPN diagnosed concomitantly in 27.6% (24/87), and CML preceding Ph-negative MPN in 23.0% (20/87) [3]. Similarly, the retrospective multi-institutional study by Soderquist et al. examining MPN with concurrent *BCR::ABL1* translocation and *JAK2* mutation identified 11 patients with 5 (45.5%) initially diagnosed with *JAK2*, 5 (45.5%) had both alterations identified simultaneously, and 1 (9.1%) initially diagnosed with *BCR::ABL1* rearrangement [9]. This was in contrast with our analysis, where the simultaneous diagnosis of CML and Ph-negative MPN was the least frequent scenario. However, only 16 of the 61 patients underwent simultaneous testing for *BCR::ABL1* and MPN driver mutations. Of these, 10 patients belonged to Group 3, with concomitant diagnoses. In most cases, testing was not performed simultaneously, and therefore a MPN or CML may have been present but not recognized at the time of initial diagnosis.

Our findings align with prior studies regarding the increased incidence of progression to myelofibrosis [3, 9] suggesting that the increasing genomic complexity involved with the combination of *BCR::ABL1* rearrangement and MPN driver mutation may contribute to this trend towards disease progression. In parallel, patients in whom Ph-negative MPN progressed to myelofibrosis also showed resistance to their TKI for CML treatment. In 18.2% (2/11) of these post-ET/PV MF, the e1a2 *BCR::ABL1* transcript was expressed, which is associated with TKI resistance and a more aggressive disease course. This raises important questions about the role of the two mutant tyrosine kinases in driving disease progression, particularly the increased frequency of myelofibrotic transformation observed in this subset of patients [9]. The coexistence of both mutations potentially contributes to, or is reflective of, a more genetically and biologically unstable hematopoietic environment. This may in turn promote clonal competition, inflammation, and ineffective hematopoiesis, which are features associated with fibrosis development. However, the underlying mechanisms remain incompletely understood, and further research is needed to clarify them.

The presence of TKI resistance across multiple international centers strengthens the generalizability of these results and underscores their relevance to diverse healthcare settings. However, it may be difficult to fully interpret TKI response due to relatively short median follow-up on first-line TKI, and absence of CHR may also be due to untreated Ph-negative MPN component. This is mainly explained by loss of response or resistance to first-line TKI, leading to early introduction of second-line TKI. Some missing data and some patients who started recently also explain the short follow-up of TKI in a few cases. This reflects the retrospective and multicenter nature of the study.

As expected, older age was associated with an adverse impact on prognosis, as was the presence of the p190 *BCR::ABL1* transcript, based on comparative analyses of overall survival between patient subgroups defined by transcript type. The lack of statistically significant difference in OS based on ELTS score, suggests that in addition to low numbers, the coexistence of the two disorders may negate the established prognostic indicators generally associated with each disease.

The interaction between *JAK2* and *BCR::ABL1* clones during disease course was described in the study by Zanelli et al. [3]. TKI treatment often led to a decline in the *BCR::ABL1* clone and an increase in the *JAK2* clone, with this observed in 81.8% of patients with concurrent disease, 65% with CML preceding MPN, and 41.9% with MPN preceding CML, which supports clonal independence. A recent study by Naumann et al. revealed, using CFU-GM analysis, that in 89% of evaluable patients, *BCR::ABL1* had emerged in a pre-existing *JAK2*-positive clone [32]. As in our analysis, atypical *BCR::ABL1* transcripts were also overrepresented at 33% as well as increased additional somatic mutations identified in 56% of subjects [32]. Clonality analysis, as performed in our study and others [28, 29], demonstrating that the conditions originate from separate hematopoietic clones, provides valuable information that could potentially inform treatment decisions. Our analysis identifying separate clonal populations was performed in two patients with CML diagnosis preceding development of Ph-negative MPN, which may explain the discrepancy in our findings compared with that of Naumann et al. [32]. in whom analysis was performed in patients with initial diagnosis of Ph-negative MPN given the recognized differences in clonal dynamics between the conditions. Clonal hierarchy may differ depending on the underlying driver mutations. For instance, *CALR* mutation is often dominant, whereas *JAK2* mutation tends to be oligoclonal. In addition, the detection of *BCR::ABL1* in *JAK2*-positive clone will be impacted in patients who achieved a DMR.

Some limitations should be acknowledged reflecting the longitudinal retrospective multi-center nature of the study. Variations in data collection methods across centers may have introduced heterogeneity, potentially influencing some outcomes. Longitudinal molecular data for the non-driver mutations were not available, and therefore clonal evolution over time could not be assessed. Clonality analysis was performed in two cases from a single center as representative examples, and broader analyses would be valuable. In addition, missing data in key variables particularly around treatment response and follow-up may impact the robustness of our analyses.

Our findings have important implications for clinical practice. The poorer outcomes, driven by increased incidence of TKI resistance and myelofibrotic transformation, emphasize the need for earlier identification, improved screening, and closer monitoring of patients with co-occurring *BCR::ABL1* rearrangement and Ph-negative MPN. We suggest testing for presence of additional MPN or CML if lack of treatment response or unexpected disease progression with atypical features, such as persistent leukocytosis or basophilia in Ph-negative MPN or persistent thrombocytosis or splenomegaly in CML. Furthermore, the detection of e1a2 *BCR::ABL1* transcript suggests that more aggressive management of these patients may improve patient outcomes. Future research should focus on exploring the underlying clonal mechanisms behind concurrent *BCR::ABL1* and MPN driver mutations. Strategies to diagnose the cases earlier should be identified to mitigate the impact of treatment resistance and myelofibrotic transformation. Larger prospective studies and collaborative efforts are essential to build on these findings and optimize care for patients with concomitant diagnosis of CML and Ph-negative MPN.

## Conclusion

In conclusion, this largest international, multicenter study provides new insights into cases with co-occurrence of *BCR::ABL1* rearrangement and Ph-negative MPN, demonstrating an increased incidence of TKI resistance and myelofibrotic transformation, in parallel with the notable presence of the e1a2 *BCR::ABL1* transcript. These findings underscore the importance of earlier recognition of this rare genetic combination and highlight the need for ongoing research to improve outcomes in this complex patient population.

## Supplementary information

Supplemental Material

## Data Availability

The datasets generated during and/or analyzed during the current study are available from the corresponding author on reasonable request.
