# Supplementary material for: Myeloproliferative neoplasms with concomitant chronic myeloid leukemia are associated with TKI resistance and poor outcomes
Source: Leukemia. 2026 Mar 27;40(5):946–54. doi: 10.1038/s41375-026-02928-z (PMC13149311; doi:10.1038/s41375-026-02928-z)
Supplement: Supplementary file 1 — Supplemental Material [file 41375_2026_2928_MOESM1_ESM.docx]

**Title: Myeloproliferative Neoplasms with Concomitant Chronic Myeloid Leukemia Are Associated with TKI Resistance and Poor Outcomes**

**SUPPLEMENTARY MATERIALS**

**Supplementary Methods**

Mononuclear cells were isolated by Ficoll-Hypaque (Sigma Aldrich, Italy) density gradient centrifugation according to manufacturer’s protocol. Successively, 1 × 105 primary mononuclear cells were seeded in MethoCult H4435 methylcellulose medium (Stemcell Technologies). Colony formation was assessed 14 days after plating. Twenty colonies for each condition were plucked, and RNA was isolated using TRIzol (Thermo Fisher Scientific). A direct reverse transcription and PCR were performed using the OneStep RT-PCR kit (Qiagen) employing the indicated forward 5′-GAGGCCTACTCATATGAACCAAAT-3′ and reverse 5′-CATGCCAACTGTTTAGCAACTTCA-3′ primers (Tirrò E. et al.). Concurrently, expression of the BCR::ABL1 transcripts was analyzed by a semi-nested PCR employing the indicated forward 5′-TATGACTGCAAATGGTACATTCC- 3′ and reverse 5′-GTTCCAACGAGCGGCTTCACT-3′ primers for the first PCR and the internal forward primer 5′-GTGCAGAGTGGAGGGAGAACA- 3′ for the second PCR. We could not detect *BCR::ABL1* transcripts in colonies expressing wild-type JAK2 (Tirrò E. et al 2019).

**Supplementary Table 1. Diagnostic Classification Systems Applied to the Study Cohort Over Time**

| Classification system | Year / edition | Key diagnostic features relevant to this study | Group 1 (n) | Group 2 (n) | Group 3 (n) | Total (n) |
| --- | --- | --- | --- | --- | --- | --- |
| French-American-British (FAB) classification | Pre-2001 | Morphology- and clinically based classification; no incorporation of molecular or cytogenetic features | 4 | 1 | 1 | 6 |
| World Health Organization (WHO) classification | 2001  (3^rd^ edition) | First WHO standard integrating morphology, cytogenetics, and clinical features; formal recognition of BCR::ABL1–positive chronic myeloid leukemia | 6 | 6 | 0 | 12 |
| WHO classification | 2008  (4^th^ edition) | Reclassification from “diseases” to “neoplasms”; incorporation of JAK2, MPL, and CALR mutations into diagnostic criteria | 16 | 3 | 3 | 22 |
| WHO classification | 2016 (revised 4^th^ edition) | Lowered hemoglobin/hematocrit thresholds for polycythemia vera; formal recognition of prefibrotic primary myelofibrosis | 1 | 2 | 2 | 5 |
| WHO classification | 2022  (5^th^ edition) | Emphasis on driver mutations; refined blast thresholds for chronic myeloid leukemia phases | 4 | 6 | 6 | 16 |

**Supplementary Table 2. Distribution of First-Line Treatment for Ph-negative MPNs in the 3 Groups**

|  | Hydroxycarbamide  (n, %) | Active surveillance, including venesections only for PV  (n, %) | Pegylated interferon  (n, %) | Ruxolitinib  (n, %) | Other  (n, %) |
| --- | --- | --- | --- | --- | --- |
| Group 1: Ph-negative MPN preceding CML  31/61 (50.8%) | 21  (67.7%) | 4  (12.9%) | 0 | 5  (16.1%) | 1  (3.2%)  (anagrelide) |
| Group 2: CML preceding Ph-negative MPN  18/61 (29.5%) | 10  (55.6%) | 5  (27.8%) | 1  (5.5%) | 2  (11.1%) | 0 |
| Group 3: Ph-negative MPN and CML diagnosed simultaneously  12/61 (19.7%) | 5  (41.7%) | 5  (41.7%) | 0 | 1  (8.3%) | 1  (8.3%)  (palliative care) |
| Overall cohort (n=61) | 36  (59.0%) | 14  (23.0%) | 1  (1.6%) | 8  (13.1%) | 2  (3.3%) |

Abbreviations: MPN: myeloproliferative neoplasm, CML: chronic myeloid leukemia, Ph: Philadelphia, PV: Polycythemia vera
